# Two-stage inertial microfluidics enrichment of activated T-cells towards a bead-less chimeric antigen receptor manufacturing protocol

**DOI:** 10.1007/s12032-026-03276-9

**Published:** 2026-01-29

**Authors:** Mona T. Elsemary, Michelle F. Maritz, Louise E. Smith, Majid Ebrahimi Warkiani, Benjamin Thierry

**Affiliations:** 1 Future Industries Institute, University of South Australia Mawson Lakes Campus, Mawson Lakes, SA 5095 Australia; 2 School of Biomedical Engineering, University of Technology Sydney, 2007 Broadway, Ultimo, NSW Australia; 3 Future Industries Institute, Building X, GPO Box 2471, Mawson Lakes, Adelaide, SA 5001 Australia

**Keywords:** Immunotherapy, Chimeric antigen receptor, Microfluidics, Ovarian cancer, T-cells

## Abstract

CAR-T cell therapy is leading the way in the field of cancer cell immunotherapies due to its high success rates. However, the manufacturing of CAR-T cells remains complex and expensive. T-cell enrichment from patient apheresis starting material is a key step in the manufacture but cellular impurities interfere with the ex vivo transduction of T-cells and their proliferation. Current enrichment methods including magnetic bead selection suffer from various limitations. We report here a bead-less T-cell enrichment process through a two-stage procedure based on inertial microfluidics. Using apheresis like starting material samples from healthy donors, the dual-stage process showed an efficient 87% (SD ± 6%) enrichment and 80% (SD ± 30%) recovery of T-cells. Validation of the process with ovarian cancer samples resulted in a T-cell purity 70% (SD ± 10%) from a starting purity of 48% (SD ± 6%) at a 64% (SD ± 4%) T-cell recovery. The two-stage inertial microfluidic process was also shown to have no detectable effect on the proliferation of the cells.

## Introduction

Chimeric Antigen Receptor (CAR) T-cell therapy is a form of adoptive cell therapy where a patient’s T-cells are genetically modified to express a cancer specific chimeric antigen receptor and then expanded ex vivo to produce the required dose for therapy. The treatment has yielded remarkably high remission rates in various types of blood cancer [1, 2]. These impressive clinical outcomes led to the approval of six CAR-T drugs to date and driven intense research in both academic and commercial settings aimed at developing new CAR-T cells and/or identifying new targets for the CARs. However, many of the well-known bottlenecks associated to the manufacturing of CAR-T products have yet to be satisfactorily addressed. CAR-T cell manufacturing has evolved since the early attempts in from investigator-led clinical protocols to centralized manufacturing facilities [3–5]. Conversely, to address the significant logistical challenges of current highly centralized manufacturing, there is a growing shift toward point-of-care manufacturing [6, 7]. This requires developing innovative methodologies that enable decentralization and automation, while ensuring the quality and reproducibility of CAR T products [8–11].

In its current autologous format, CAR-T cell therapy relies on a patient own T-cells that are enriched at the start of the manufacturing process. Significant variation in the composition of the initial apheresis product in regard to the amount of T-cells and their purity is now well documented, which is a major obstacle to standardization of the manufacturing process and occasionally even lead to manufacture failure [12]. Specifically, the T-cells percentages in apheresis products range anywhere from 10% to 90% depending on the patient’s state, type of cancer, cancer stage and prior treatments. Several methods to enrich either the lymphocyte fraction or T-cells have been developed. This includes centrifugation based methods such as elutriation [13] and Ficoll-Paque gradient centrifugation [14], and non-centrifugation based methods such as positive or negative immunomagnetic selection [15] and acoustic wave-based methods [16]. Immunomagnetic selection with magnetic beads yields the highest T-cell purity (ranging from 85% to 93% [13, 17–19]. However, immunomagnetic selection suffers from several drawbacks including modification of the phenotype and activation levels [20], low throughput and recovery [13, 18], and the need for bead removal [21]. There is therefore a need for better T-cells enrichment technology.

We have previously demonstrated that microfluidic processing can efficiently enrich lymphocytes from leukapheresis products by depleting larger white blood cells. We also found that, owing to the enlarged size of B-cell blasts in Acute Lymphoblastic Leukemia (ALL) blood, inertial microfluidics can significantly enrich T-cells with minimal impact on their function and phenotype [22]. More generally, inertial microfluidic devices have been developed to achieve size-based fractionation of cellular suspensions and were successfully applied to the enrichment of circulating cancer cells [23], white blood cells [24], lymphocytes [22] and circulating fetal cells [25]. Spiral inertial microfluidic fractionation is based on the principle that in laminar flow in spiral channels two dominant forces (lift and dean drag forces) act on suspended cells/particles. Larger cells tend to experience stronger lift forces than smaller ones, the latter being more influenced by the dean drag forces [26, 27]. Microfluidic technologies have already been implemented in the field of CAR-T cell manufacturing including the CURATE™ chip for cell separation [28, 29] and Draper chip for transduction [30, 31]. Microfluidic technologies have the potential to support GMP-compliant processes that could be operated in non-clean room facilities [32, 33], aiding in the decentralization of adoptive cell therapy manufacturing. Alternatively, they can be integrated into fully enclosed robotic platforms, such as those used by Cellares or Ori Biotech [34, 35].

**Fig. 1:**
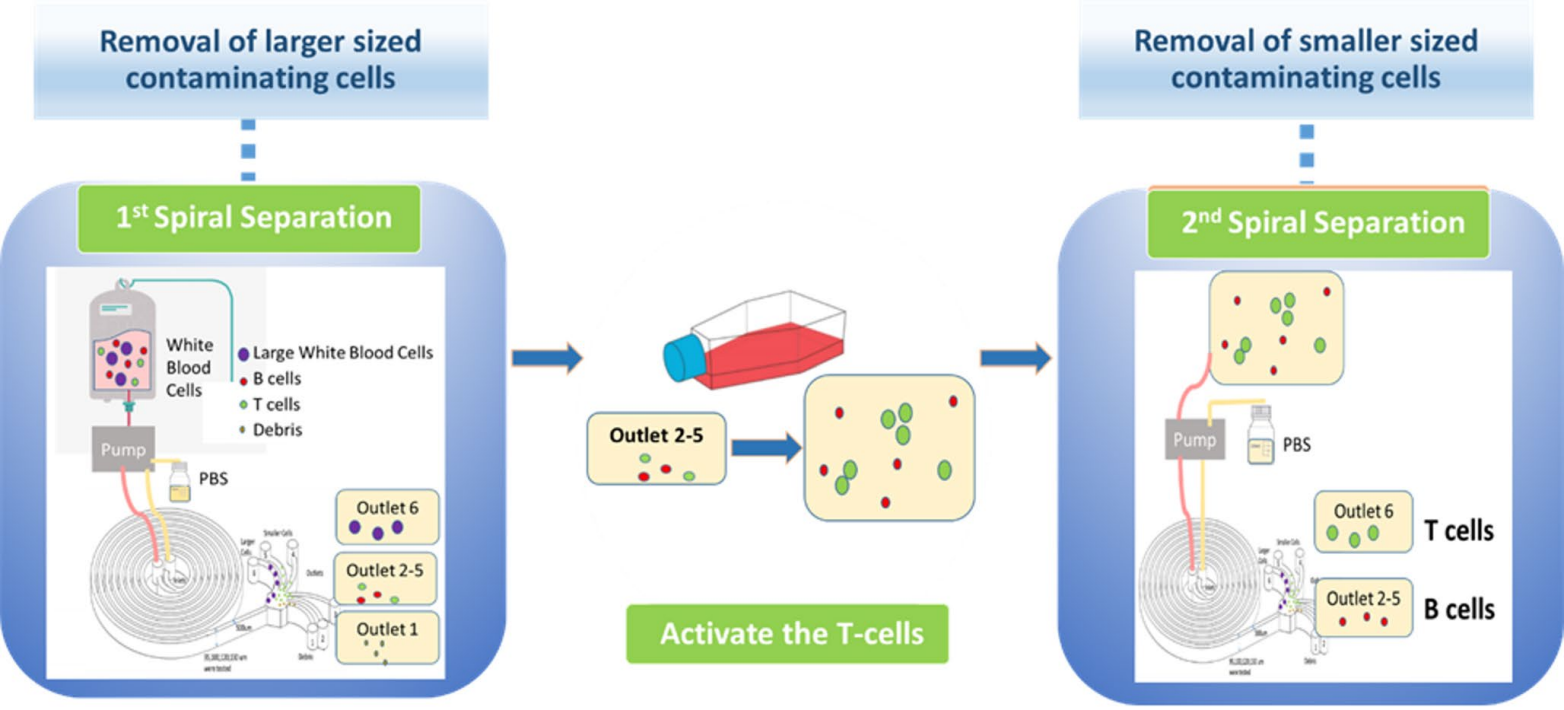
Schematic representation of the two-step inertial microfluidic enrichment of activated T-cells. Larger contaminating cells (monocytes, granulocytes, large leukemic blasts) are depleted in the first spiral separation and smaller contaminating cells (B-cells and other smaller white blood cells) are depleted in the second spiral separation to enrich enlarged activated T-cells

In our previous work, purities of 91% for lymphocytes and 73% for T-cells were achieved after a single separation inertial microfluidic enrichment step for ALL patient donor samples. However, this approach took advantage of the relatively large size of cancerous blasts to efficiently enrich the T-cells fraction in a single size fractionation step. This approach provides little scope beyond ALL and other blood cancer where large B-cell blasts predominate namely acute myeloid leukemia [36], diffuse large B-cell lymphoma and other non-Hodgkin lymphomas [37]. Towards broadening the application of inertial microfluidics to other types of cancer including solid ones, we therefore propose and demonstrate here a two-step inertial microfluidic T-cell enrichment methodology where the lymphocyte fraction is enriched in the first step and the activated T-cells are enriched in the second step owing to their increased size [38–40]. Activation is an integral part of the CAR-T cell manufacturing process required for efficient T-cell transduction and ex vivo proliferation. Upon activation, T-cells exhibit a different size distribution profile which provides here the impetus for their separation from smaller B lymphocytes and to some extent non-activated T-cells **(**Fig. 1**)**. Efficient enrichment of T-cells was achieved in ovarian cancer patient donor samples.

## Materials and methods

### Device design, fabrication and operation

The spiral microfluidic device used here was optimized previously [22, 43]. The polydimethylsiloxane (PDMS) microchannel devices were fabricated using standard soft lithography from a SU-8 mold which was designed and fabricated by photolithography at the Australian National Fabrication Facility. Briefly, the devices were prepared by mixing the base and curing agent (Sylgard 184, Dow Corning Inc.) in a 10:1 ratio, pouring into the mold, degassing and then curing at 60 °C. The cured devices were released from the molds and inlet/outlet ports were punched using a 14-gauge puncher and then bonded to glass slides using air plasma (Harrick Plasma, USA). The device used in both separation steps was a low aspect ratio eight turn spiral microchannel device comprised of two inlets and six outlets. The microchannel width is 500 μm and its height 130 μm. Different microchannel heights were initially tested to modulate the flow conditions ranging from 95 to 130 μm. Two 15 cm inlet tubings were attached to the device and a syringe pump. Outlet tubings for the six outlets were 5 cm each, except for the tubing connected to the outlet 6, which was 15 cm.Prior to cell separation, devices were treated with 1% bovine serum albumin (BSA) to minimize non-specific fouling as previously reported [44]. Cells were suspended in phosphate buffered saline (PBS) + 1% fetal bovine serum (FBS) (Gibco, USA). Different operation conditions were optimized by us.

Different flow rate ratios were previously tested in our lab and the effect on separation efficiency investigated [43]. The flow rate that was selected was 1 mL/min in both inlets for the first separation and second separation. The greatest separation (size cut off value) was observed between outlet 5 and 6 and thus cells collected from outlet 2–5 were pooled. In outlet 1, debris and smaller sized platelets (average 2 μm) are present and therefore discarded. The size cutoff between the recovered and waste streams was 7 μm. Most cells smaller than 7 μm were collected in outlets 2–5 whereas cells larger than 7 μm were mostly collected in outlet 6. After the first separation in the device, T-cells, B-cells and other smaller white blood cells were collected from the outlets. Following the first separation, an activation step was integrated to allow for an increase in T-cell size to enable separation from the other cells in a second device separation step (as shown in Fig. 1). After T-cell activation, a second separation step of the leukocytes was performed to separate the enlarged activated T-cells from the smaller cell impurities (B-cells and other white blood cells) at a flow rate of 1 mL/min.

The recovery and purity of the different cell types in the collection outlets 2–5 were calculated as follows:

1 $$\begin{aligned}\:\mathrm{P}\mathrm{e}\mathrm{r}\mathrm{c}\mathrm{e}\mathrm{n}\mathrm{t}\mathrm{a}\mathrm{g}\mathrm{e}\:\mathrm{R}\mathrm{e}\mathrm{c}\mathrm{o}\mathrm{v}\mathrm{e}\mathrm{r}\mathrm{y}\:\mathrm{o}\mathrm{f}\:\mathrm{t}\mathrm{h}\mathrm{e}\:\mathrm{C}\mathrm{e}\mathrm{l}\mathrm{l}\:\mathrm{T}\mathrm{y}\mathrm{p}\mathrm{e}\:\mathrm{i}\mathrm{n}\:\mathrm{f}\mathrm{i}\mathrm{r}\mathrm{s}\mathrm{t}\:\mathrm{s}\mathrm{e}\mathrm{p}\mathrm{a}\mathrm{r}\mathrm{a}\mathrm{t}\mathrm{i}\mathrm{o}\mathrm{n}\\=\frac{\mathrm{S}\mathrm{u}\mathrm{m}\:\mathrm{o}\mathrm{f}\:\mathrm{c}\mathrm{e}\mathrm{l}\mathrm{l}\:\mathrm{n}\mathrm{u}\mathrm{m}\mathrm{b}\mathrm{e}\mathrm{r}\:\mathrm{o}\mathrm{f}\:\mathrm{t}\mathrm{h}\mathrm{e}\:\mathrm{C}\mathrm{e}\mathrm{l}\mathrm{l}\:\mathrm{T}\mathrm{y}\mathrm{p}\mathrm{e}\:\mathrm{i}\mathrm{n}\:\mathrm{O}\mathrm{u}\mathrm{t}\mathrm{l}\mathrm{e}\mathrm{t}\mathrm{s}\:2-5}{\mathrm{S}\mathrm{u}\mathrm{m}\:\mathrm{o}\mathrm{f}\:\mathrm{c}\mathrm{e}\mathrm{l}\mathrm{l}\:\mathrm{n}\mathrm{u}\mathrm{m}\mathrm{b}\mathrm{e}\mathrm{r}\:\mathrm{o}\mathrm{f}\:\mathrm{t}\mathrm{h}\mathrm{e}\:\mathrm{C}\mathrm{e}\mathrm{l}\mathrm{l}\:\mathrm{T}\mathrm{y}\mathrm{p}\mathrm{e}\:\mathrm{i}\mathrm{n}\:\mathrm{a}\mathrm{l}\mathrm{l}\:\mathrm{o}\mathrm{u}\mathrm{t}\mathrm{l}\mathrm{e}\mathrm{t}\mathrm{s}}{\times}\:100\end{aligned}$$

2 $$\begin{aligned}\:\mathrm{P}\mathrm{e}\mathrm{r}\mathrm{c}\mathrm{e}\mathrm{n}\mathrm{t}\mathrm{a}\mathrm{g}\mathrm{e}\:\mathrm{P}\mathrm{u}\mathrm{r}\mathrm{i}\mathrm{t}\mathrm{y}\:\mathrm{o}\mathrm{f}\:\mathrm{t}\mathrm{h}\mathrm{e}\:\mathrm{C}\mathrm{e}\mathrm{l}\mathrm{l}\:\mathrm{T}\mathrm{y}\mathrm{p}\mathrm{e}\:\mathrm{i}\mathrm{n}\:\mathrm{f}\mathrm{i}\mathrm{r}\mathrm{s}\mathrm{t}\:\mathrm{s}\mathrm{e}\mathrm{p}\mathrm{a}\mathrm{r}\mathrm{a}\mathrm{t}\mathrm{i}\mathrm{o}\mathrm{n}\\\:=\frac{\mathrm{S}\mathrm{u}\mathrm{m}\:\mathrm{o}\mathrm{f}\:\mathrm{c}\mathrm{e}\mathrm{l}\mathrm{l}\:\mathrm{n}\mathrm{u}\mathrm{m}\mathrm{b}\mathrm{e}\mathrm{r}\:\mathrm{o}\mathrm{f}\:\mathrm{t}\mathrm{h}\mathrm{e}\:\mathrm{C}\mathrm{e}\mathrm{l}\mathrm{l}\:\mathrm{T}\mathrm{y}\mathrm{p}\mathrm{e}\:\mathrm{i}\mathrm{n}\:\mathrm{O}\mathrm{u}\mathrm{t}\mathrm{l}\mathrm{e}\mathrm{t}\mathrm{s}\:2-5}{\mathrm{S}\mathrm{u}\mathrm{m}\:\mathrm{o}\mathrm{f}\:\mathrm{c}\mathrm{e}\mathrm{l}\mathrm{l}\:\mathrm{n}\mathrm{u}\mathrm{m}\mathrm{b}\mathrm{e}\mathrm{r}\:\mathrm{o}\mathrm{f}\:\mathrm{a}\mathrm{l}\mathrm{l}\:\mathrm{C}\mathrm{e}\mathrm{l}\mathrm{l}\mathrm{s}\:\mathrm{i}\mathrm{n}\:\:\mathrm{O}\mathrm{u}\mathrm{t}\mathrm{l}\mathrm{e}\mathrm{t}\mathrm{s}\:2-5}{\times}\:100\end{aligned}$$

3 $$\begin{aligned}\:\mathrm{P}\mathrm{e}\mathrm{r}\mathrm{c}\mathrm{e}\mathrm{n}\mathrm{t}\mathrm{a}\mathrm{g}\mathrm{e}\:\mathrm{R}\mathrm{e}\mathrm{c}\mathrm{o}\mathrm{v}\mathrm{e}\mathrm{r}\mathrm{y}\:\mathrm{o}\mathrm{f}\:\mathrm{t}\mathrm{h}\mathrm{e}\:\mathrm{C}\mathrm{e}\mathrm{l}\mathrm{l}\:\mathrm{T}\mathrm{y}\mathrm{p}\mathrm{e}\:\mathrm{i}\mathrm{n}\:\mathrm{s}\mathrm{e}\mathrm{c}\mathrm{o}\mathrm{n}\mathrm{d}\:\mathrm{s}\mathrm{e}\mathrm{p}\mathrm{a}\mathrm{r}\mathrm{a}\mathrm{t}\mathrm{i}\mathrm{o}\mathrm{n}\\=\frac{\mathrm{S}\mathrm{u}\mathrm{m}\:\mathrm{o}\mathrm{f}\:\mathrm{c}\mathrm{e}\mathrm{l}\mathrm{l}\:\mathrm{n}\mathrm{u}\mathrm{m}\mathrm{b}\mathrm{e}\mathrm{r}\:\mathrm{o}\mathrm{f}\:\mathrm{t}\mathrm{h}\mathrm{e}\:\mathrm{C}\mathrm{e}\mathrm{l}\mathrm{l}\:\mathrm{T}\mathrm{y}\mathrm{p}\mathrm{e}\:\mathrm{i}\mathrm{n}\:\mathrm{O}\mathrm{u}\mathrm{t}\mathrm{l}\mathrm{e}\mathrm{t}\mathrm{s}\:6}{\mathrm{S}\mathrm{u}\mathrm{m}\:\mathrm{o}\mathrm{f}\:\mathrm{c}\mathrm{e}\mathrm{l}\mathrm{l}\:\mathrm{n}\mathrm{u}\mathrm{m}\mathrm{b}\mathrm{e}\mathrm{r}\:\mathrm{o}\mathrm{f}\:\mathrm{t}\mathrm{h}\mathrm{e}\:\mathrm{C}\mathrm{e}\mathrm{l}\mathrm{l}\:\mathrm{T}\mathrm{y}\mathrm{p}\mathrm{e}\:\mathrm{i}\mathrm{n}\:\mathrm{a}\mathrm{l}\mathrm{l}\:\mathrm{o}\mathrm{u}\mathrm{t}\mathrm{l}\mathrm{e}\mathrm{t}\mathrm{s}}\times\:100\end{aligned}$$

4 $$\begin{aligned}\:\mathrm{P}\mathrm{e}\mathrm{r}\mathrm{c}\mathrm{e}\mathrm{n}\mathrm{t}\mathrm{a}\mathrm{g}\mathrm{e}\:\mathrm{P}\mathrm{u}\mathrm{r}\mathrm{i}\mathrm{t}\mathrm{y}\:\mathrm{o}\mathrm{f}\:\mathrm{t}\mathrm{h}\mathrm{e}\:\mathrm{C}\mathrm{e}\mathrm{l}\mathrm{l}\:\mathrm{T}\mathrm{y}\mathrm{p}\mathrm{e}\:\mathrm{i}\mathrm{n}\:\mathrm{s}\mathrm{e}\mathrm{c}\mathrm{o}\mathrm{n}\mathrm{d}\:\mathrm{s}\mathrm{e}\mathrm{p}\mathrm{a}\mathrm{r}\mathrm{a}\mathrm{t}\mathrm{i}\mathrm{o}\mathrm{n}\\\:=\frac{\mathrm{S}\mathrm{u}\mathrm{m}\:\mathrm{o}\mathrm{f}\:\mathrm{c}\mathrm{e}\mathrm{l}\mathrm{l}\:\mathrm{n}\mathrm{u}\mathrm{m}\mathrm{b}\mathrm{e}\mathrm{r}\:\mathrm{o}\mathrm{f}\:\mathrm{t}\mathrm{h}\mathrm{e}\:\mathrm{C}\mathrm{e}\mathrm{l}\mathrm{l}\:\mathrm{T}\mathrm{y}\mathrm{p}\mathrm{e}\:\mathrm{i}\mathrm{n}\:\mathrm{O}\mathrm{u}\mathrm{t}\mathrm{l}\mathrm{e}\mathrm{t}\:6}{\mathrm{S}\mathrm{u}\mathrm{m}\:\mathrm{o}\mathrm{f}\:\mathrm{c}\mathrm{e}\mathrm{l}\mathrm{l}\:\mathrm{n}\mathrm{u}\mathrm{m}\mathrm{b}\mathrm{e}\mathrm{r}\:\mathrm{o}\mathrm{f}\:\mathrm{a}\mathrm{l}\mathrm{l}\:\mathrm{C}\mathrm{e}\mathrm{l}\mathrm{l}\mathrm{s}\:\mathrm{i}\mathrm{n}\:\:\mathrm{O}\mathrm{u}\mathrm{t}\mathrm{l}\mathrm{e}\mathrm{t}\:6}\times\:100\end{aligned}$$

5 $$\begin{aligned}\:\mathrm{O}\mathrm{v}\mathrm{e}\mathrm{r}\mathrm{a}\mathrm{l}\mathrm{l}\:\mathrm{R}\mathrm{e}\mathrm{c}\mathrm{o}\mathrm{v}\mathrm{e}\mathrm{r}\mathrm{y}\:\mathrm{f}\mathrm{o}\mathrm{r}\:\mathrm{t}\mathrm{h}\mathrm{e}\:\mathrm{t}\mathrm{w}\mathrm{o}\\-\mathrm{s}\mathrm{t}\mathrm{e}\mathrm{p}\:\mathrm{p}\mathrm{r}\mathrm{o}\mathrm{c}\mathrm{e}\mathrm{s}\mathrm{s}=\mathrm{\%}\:\mathrm{R}\mathrm{e}\mathrm{c}\mathrm{o}\mathrm{v}\mathrm{e}\mathrm{r}\mathrm{y}\:\mathrm{i}\mathrm{n}\:\mathrm{f}\mathrm{i}\mathrm{r}\mathrm{s}\mathrm{t}\:\mathrm{s}\mathrm{e}\mathrm{p}\mathrm{a}\mathrm{r}\mathrm{a}\mathrm{t}\mathrm{i}\mathrm{o}\mathrm{n}\\\times\:\mathrm{\%}\:\mathrm{R}\mathrm{e}\mathrm{c}\mathrm{o}\mathrm{v}\mathrm{e}\mathrm{r}\mathrm{y}\:\mathrm{i}\mathrm{n}\:\mathrm{s}\mathrm{e}\mathrm{c}\mathrm{o}\mathrm{n}\mathrm{d}\:\mathrm{s}\mathrm{e}\mathrm{p}\mathrm{a}\mathrm{r}\mathrm{a}\mathrm{t}\mathrm{i}\mathrm{o}\mathrm{n}\:\:\:\\\times\:\:\mathrm{P}\mathrm{r}\mathrm{o}\mathrm{l}\mathrm{i}\mathrm{f}\mathrm{e}\mathrm{r}\mathrm{a}\mathrm{t}\mathrm{i}\mathrm{o}\mathrm{n}\:\mathrm{I}\mathrm{n}\mathrm{d}\mathrm{e}\mathrm{x}\:\mathrm{o}\mathrm{v}\mathrm{e}\mathrm{r}\:\mathrm{t}\mathrm{h}\mathrm{e}\:\mathrm{t}\mathrm{w}\mathrm{o}\:\mathrm{d}\mathrm{a}\mathrm{y}\:\mathrm{p}\mathrm{e}\mathrm{r}\mathrm{i}\mathrm{o}\mathrm{d}\end{aligned}$$

Proliferative index was calculated for each donor by counting the number of cells at day 2 after activation and dividing it by the initial number of cells. The mean proliferative index for healthy donors in our experiments was 1.55 (SD + 0.1) and 1.35 (SD + 0.4) for ovarian cancer patients.

### Leukocyte separation from ovarian cancer patient donor blood samples

Ovarian cancer donor blood samples were obtained from the department of Gynecological Oncology at the Royal Adelaide Hospital. Blood from healthy and ovarian cancer patient donors was collected in compliance with the University of South Australia Human Research Ethics Committee (protocol number: 201980 and 201777) and first separated using Ficoll Paque Premium (GE Healthcare, USA) according to the manufacturer instructions. The blood was diluted with equal volume of Dulbecco Modified PBS and then layered carefully on top of the Ficoll Paque medium. The tube was centrifuged at 400 × g for 40 min at 20 °C. Two layers were collected, the mononuclear layer at the interface of the plasma and the Ficoll Paque and the granulocyte layer, located as thin layer above the red blood cell (RBC) pellet. This mixture was used to mimic apheresis products. The cell suspensions were transferred to new tubes and washed with PBS + 1% FBS.

### Cell culture

White blood cells from healthy and patient donors (enriched or not) were cultured in complete RPMI medium (RPMI 1640 medium, 10% FBS, 1% glutamate, 1% penicillin/streptomycin) (Life technologies, Australia) at 37 °C in a humidified atmosphere containing 5% (v/v) CO_2_.

### T-cell activation

T-cell activation was optimized by testing various combinations of CD3 and CD28 antibodies while maintaining a constant IL-2 concentration of 500 IU/mL (Life Technologies, Australia). The different activation combinations tested were 5/4, 5/2, 3/4, 3/2, 1/4 and 1/2 µg/mL of anti-CD3/anti-CD28. Microtiter plates were first coated with CD3 antibody overnight (50 µL per well in a 96 well plate) at 4 °C. The next day the plates were washed with PBS and the cells were seeded at a concentration of 1 × 10^5^ cells/mL in 200 µL complete RPMI medium containing various anti-CD28 concentrations and IL-2. The cells were then tested for CD69 expression used as a T cell activation marker and size increases after 2 and 3 days were measured using imaging flow cytometry. The activation regimen used in subsequent experiments was selected based on the expression of CD69 and T-cell size increase.

### Flow cytometry

Cells were stained for surface markers and visualized using an ImageStreamx Mark II imaging flow cytometer (AMNIS, Seattle, WA, USA). Cells recovered from patient samples were stained with anti-human CD45 EF450 (clone HI30), T-cells with anti-human CD3 FITC (clone SK7), B-cells with anti-human CD19 APC (clone HIB19) (Life Technologies, Australia). For measuring activation level cells were stained for the early activation marker CD69 using anti-human CD69 PE (clone FN50). Phenotyping was done by staining for antihuman CD45RO PE (clone UCHL1), CD45RA PerCP-CY 5.5 (clone HI 100) and CCR7 BV421 (clone 150503) (BD Life Sciences, Australia). Analysis of the cellular populations was performed with the IDEAS software Version 6.1 (AMNIS, Seattle, WA, USA) and FlowJo V10 (FLOWJO, USA). Staining was done in siliconized polypropylene tubes after resuspending the cell pellets in ice cold PBS, 10% FBS and 1% sodium azide at a concentration of 2 × 10^7^ cells/mL. 0.1–10 µg/mL of the primary labelled antibody was added and then incubated for 45 min at room temperature, after which the cells were washed and suspended in ice cold PBS, 10% FBS and 1% sodium azide and used for flow cytometry. Cell size distributions was calculated using an adaptive erode mask (M04 CH04 77) on the AMNIS software, which allows a more accurate calculation of cell diameters. Unstained, single stained and Fluorescence Minus One (FMOs) control were used for compensation and adjustment of gates.

### Cell viability and proliferation

Leukocytes viability was assessed using PI exclusion staining using standard protocols. Cell proliferation was determined by staining with the proliferation dye Carboxyfluorescein succinimidyl ester (CFSE) (Biolegend, Australia). Cells were suspended at 2 × 10^6^ cells/mL in PBS and labeled with 10 µM CFSE for 10 min at 37 °C. The reaction was stopped by adding an equal volume of FBS and incubation for 2 min at room temperature. The cells were then washed twice, and the CFSE-labeled cells were cultured at 37 °C and 5% CO_2_ in 96-well microtiter plates in complete RPMI medium + 500IU/mL IL-2. Proliferation was compared between the non-enriched leukocytes and the enriched leukocytes after separation over 72 h at 24 h intervals.

### Statistical analysis

All experiments were repeated at least three times and analyzed non-parametrically using GraphPad Prism. For single factor experiments, non-parametric paired signed rank Wilcoxon T-test was carried out and for multiple factor experiments; non-parametric two- way ANOVA (mixed model for matched values) was carried out. All statistical analysis is carried out at *p* < 0.05.

## Results

### Enrichment of lymphocytes from leukocytes of normal healthy donors

**Fig. 2:**
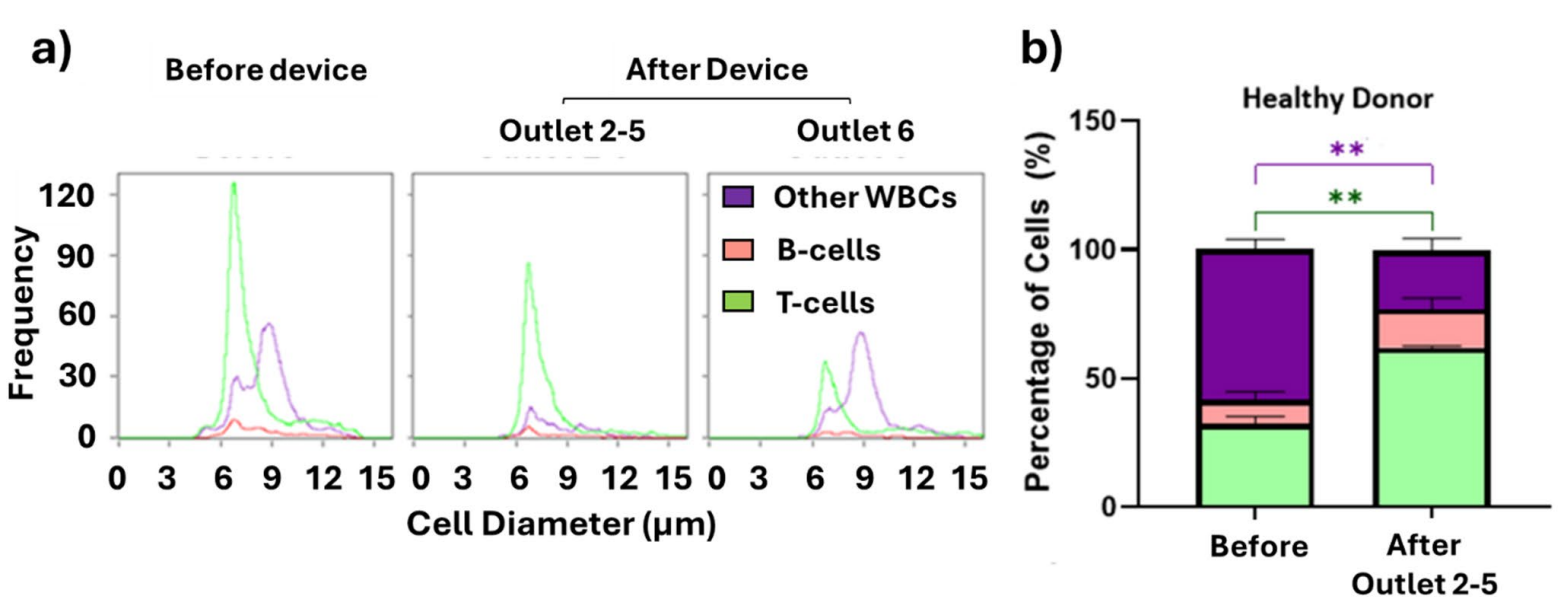
Enrichment of T-cells from healthy donor leukocytes. (**a**) Representative size distribution histograms of T-cells (green), B-cells (red) and other white blood cells (purple) before and after inertial microfluidic separation. (**b**) Cell percentages before and after inertial microfluidic separation (*n* = 7, ** *p* < 0.005 non-parametric two-way mixed model ANOVA for matched values was used followed by Bonferroni’s multiple comparisons test)

We have previously demonstrated the feasibility of enriching lymphocytes from white blood cell samples using an inertial microfluidic device. The eight-turn inertial spiral microfluidic device separates cells based on their size and enable efficient depletion of larger cells including monocytes and granulocytes. The smaller cellular fractions, mainly T-cells and B-cells are collected in outlets 2–5, while most larger leukocytes (monocytes, granulocytes and other large lymphocytes) are collected in outlet 6 whilst debris were collected in outlet 1 (Fig. 2). Under optimized conditions, inertial microfluidic achieves in healthy blood donor lymphocyte purities of 91% (SD ± 6%) from a starting purity of 65% (SD ± 20%) and a T-cell purity of 73% (SD ± 2%) from a starting purity of 45% (SD ± 10%) with recoveries of 63% (SD ± 4%) and 60% (SD ± 22%) for lymphocytes and T-cells respectively.

### Optimization of T cell activation

The one-step inertial microfluidic size fractionation process has no detectable detrimental effects on the viability and functionality of the cells and therefore enables a simple enrichment of lymphocytes. However, at the exception of cancers characterized by the presence of enlarged B-cell blasts such as in B-cell acute lymphoblastic leukemia, acute myeloid leukemia, diffuse large B-cell lymphoma and other non-Hodgkin lymphomas, T-cells and B-cells have similar size distribution and are therefore collected in the same outlets (2–5) of the inertial microfluidic device. We posit that activation of T-cells will significantly increase their size, and thereby provide a mechanism for their separation from B-cells and other small cells using a second inertial microfluidic step. It is noteworthy that T-cell activation is an essential step in CAR-T cell manufacturing that serves two main purposes; first, it improves the transduction rate and second, it stimulates their expansion which is a key requirement to reach the dose required for treating patients [5]. Following activation, T-cells increase in size as they prepare themselves for division [38, 39]. Activation also results in the increased expression of the early activation marker CD69 [45].

We first assessed the effect of several T-cell specific activation regimen based on immobilized anti-CD3 and soluble anti-CD28 antibodies. Activation using lipopolysaccharides (LPS), phorbol myristate acetate (PMA) and ionomycin were not used as they may lead to activation of B-cells and other white blood cells[46–48].

**Fig. 3:**
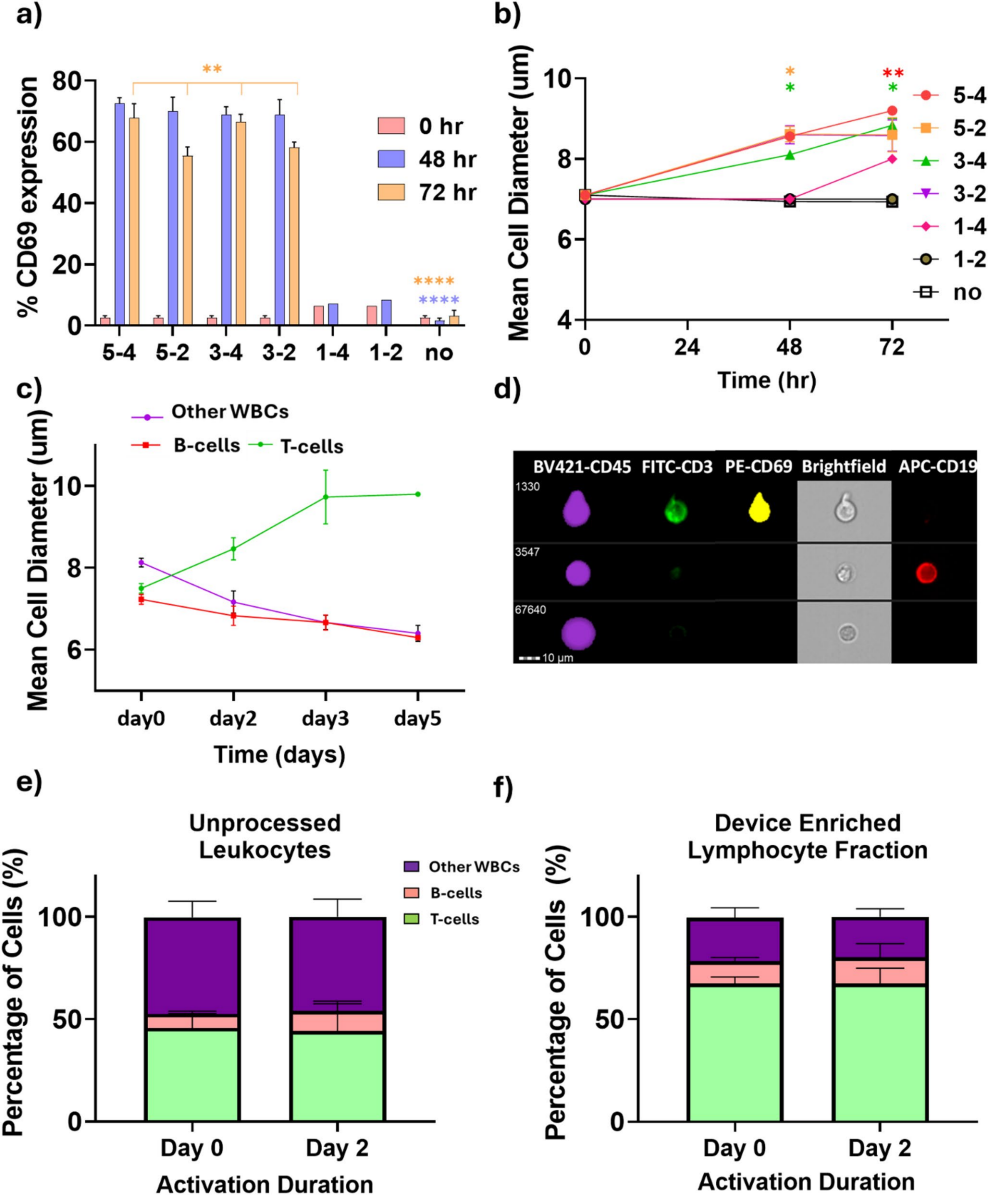
T cells size increases vs. activation. (**a**) CD69 expression assessed by imaging flow cytometry vs. T-cells activation regimen. (**b**) T-cells sizes vs. activation regimen. (**c**) Evolution of size vs. time post-activation for T-cells, B-cells, and other white blood cells in healthy donor samples (**d**) Composite images (bright field, anti-CD3, anti-CD19 and anti-CD69 surface marker staining) of T-cells, B-cells and other white blood cells after 2-day activation. Percentage of T-cells, B-cells and other white blood cells at day 0 before and at day 2 after activation with 5/4 µg/mL of anti-CD3/anti-CD28 for (**e**) Unprocessed leukocytes (**f**) Device enriched lymphocyte fraction. (*n* = 7, *p < 0.05, ** *p* < 0.005, **** *p* < 0.00005 non-parametric two- way mixed model ANOVA for matched values was used followed by Bonferroni’s multiple comparisons test)

To this end, isolated T-cells were activated with different combinations of CD3 and CD28 concentrations and the size changes and CD69 expression over time were determined. The regimens with higher concentrations of activating antibodies (5/4, 5/2, 3/4, 3/2 µg/mL of anti-CD3/anti-CD28), resulted in significantly higher activation, ranging between 56% and 73%, based on CD69 expression at both 48 and 72 h, compared to regimens with lower antibodies (1/4 and 1/2 µg/mL of anti-CD3/anti-CD28) and no antibody (Fig. 3a). As expected, the T-cell diameters increased over time upon activation (Fig. 3b). For example, a statistically significant increase was observed for the 5/4 µg/mL of anti-CD3/anti-CD28 activation regimen as compared to no increase in the absence of activation, from an average of 7.1 μm (SD ± 0.09) to 8.5 μm (SD ± 0.4) at 48 h and 9.2 μm (SD ± 0.2) at 72 h. This activation regimen was selected and used subsequently.

We next assessed the activation induced cell size changes for T-cells in the presence of B-cells and other cells by activating the entire PBMC population from healthy donors. At 48 h, T-cells increased in size from 7.5 μm (SD ± 0.2) to 8.5 μm (SD ± 0.5) (statistically significant at *p* < 0.05), while B-cell sizes decreased from 7.2 μm (SD ± 0.2) to 6.8 μm (SD ± 0.4) (not significant, *p* > 0.05). At 72 h, T-cells increased to 9.7 μm (SD ± 1.1) (statistically significant at *p* < 0.0005), while B-cell remained constant at 6.7 μm (SD ± 0.3) (Fig. 3c, d). The increase in T-cells sizes was statistically significant vs. B-cells (*p* < 0.05 at 48 h, *p* < 0.0005 at 72 h) as shown in Fig. 3c.

Finally, we assessed the cellular composition before and after the two-day activation. No significant changes were observed for both samples processed through the inertial microfluidic device (lymphocyte enriched population in first separation step) and untreated ones (Fig. 3e, f). For the unprocessed healthy donor leukocyte samples, the percentages after two-day activation of were 44% (vs. 46% at day 0) for T-cells, 10% (vs. 7% at day 0) for B-cells and 46% (vs. 47% at day 0) for other white blood cells (Fig. 3e). Similarly, lymphocyte fraction enriched in the inertial microfluidic device, the percentage of T-cells following the two-day activation was 68% (vs. 67% at day 0), B-cells was 13% (vs. 11% at day 0) and other white blood cells was 20% (vs. 21% at day 0) (Fig. 3f).

### Enrichment of activated T cells with inertial microfluidics

**Fig. 4:**
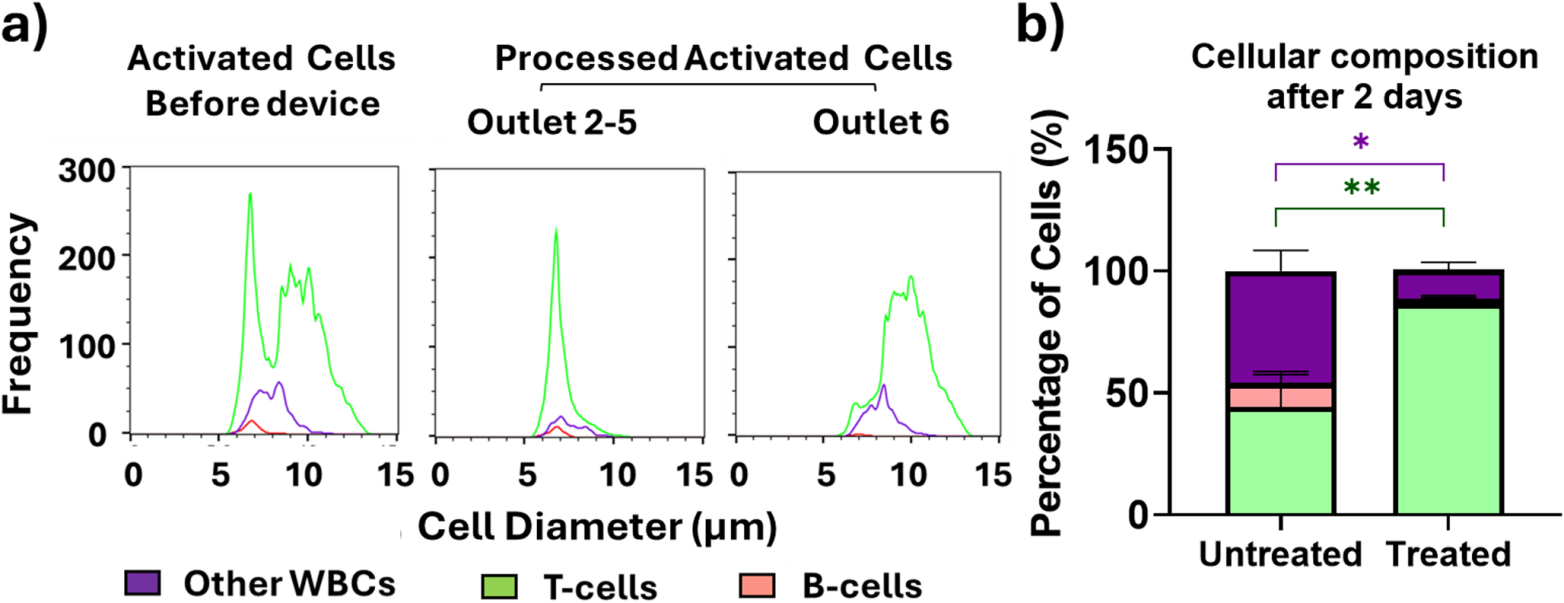
(**a**) Representative histograms of size distributions before and after inertial microfluidic separation following activation with 5/4 µg/mL of anti-CD3/anti-CD28: T-cells (green), B-cells (red) and other WBCs (violet). (**b**) Percentage of T-cells, B-cells and other white blood cells at day 2 after activation with and without inertial microfluidic separation. (*n* = 7, *p < 0.05, ** *p* < 0.005, **** *p* < 0.00005 non-parametric two- way mixed model ANOVA for matched values was used followed by Bonferroni’s multiple comparisons test)

The second separation step was carried at 48 h post-activation rather than 72 h even though the latter yielded better separation efficacy (data not shown). The reason for this is that most viral transduction protocols are carried out 24–72 h after activation with lentiviral transfection typically performed at 24–48 h [49, 50]. As expected, the lack of changes in the relative percentage of T-cells, B-cells or other white blood cells following the two-day activation period indicates that these contaminants persist after T-cells activation and hence are present at substantial concentration during the transfection/transduction processes. For the second separation step, we anticipate the feasibility of enriching the enlarged activated T-cells in outlet 6, while depleting smaller cell contaminants including B-cells in outlets 2–5. This hypothesis was first tested in healthy donor samples using the activated lymphocyte fraction obtained after the first inertial microfluidic step. As expected, B-lymphocytes (6.4 μm SD ± 0.3) were efficiently collected in outlets 2–5 (at a recovery of 74% SD ± 5%). This resulted in a significant enrichment of T-cells in outlet 6 with an increased purity of 87% (SD ± 6%) vs. 68% (SD + 17%) as shown in Fig. 4, and a recovery of 75% (SD ± 0.05) was obtained. Overall, for the complete two-step process, the recovery of T-cells was 80% (SD ± 30%) and the final T-cell purity was enhanced to 87% (SD ± 5%) compared to 44% (SD ± 22%) in the untreated leukocytes (Fig. 4b). This performance can be compared to one step elutriation, where a 70% T-cell purity from an initial purity of 61% was previously reported for healthy blood [51].

### Enrichment of activated T cells in ovarian cancer patients’ samples

**Fig. 5:**
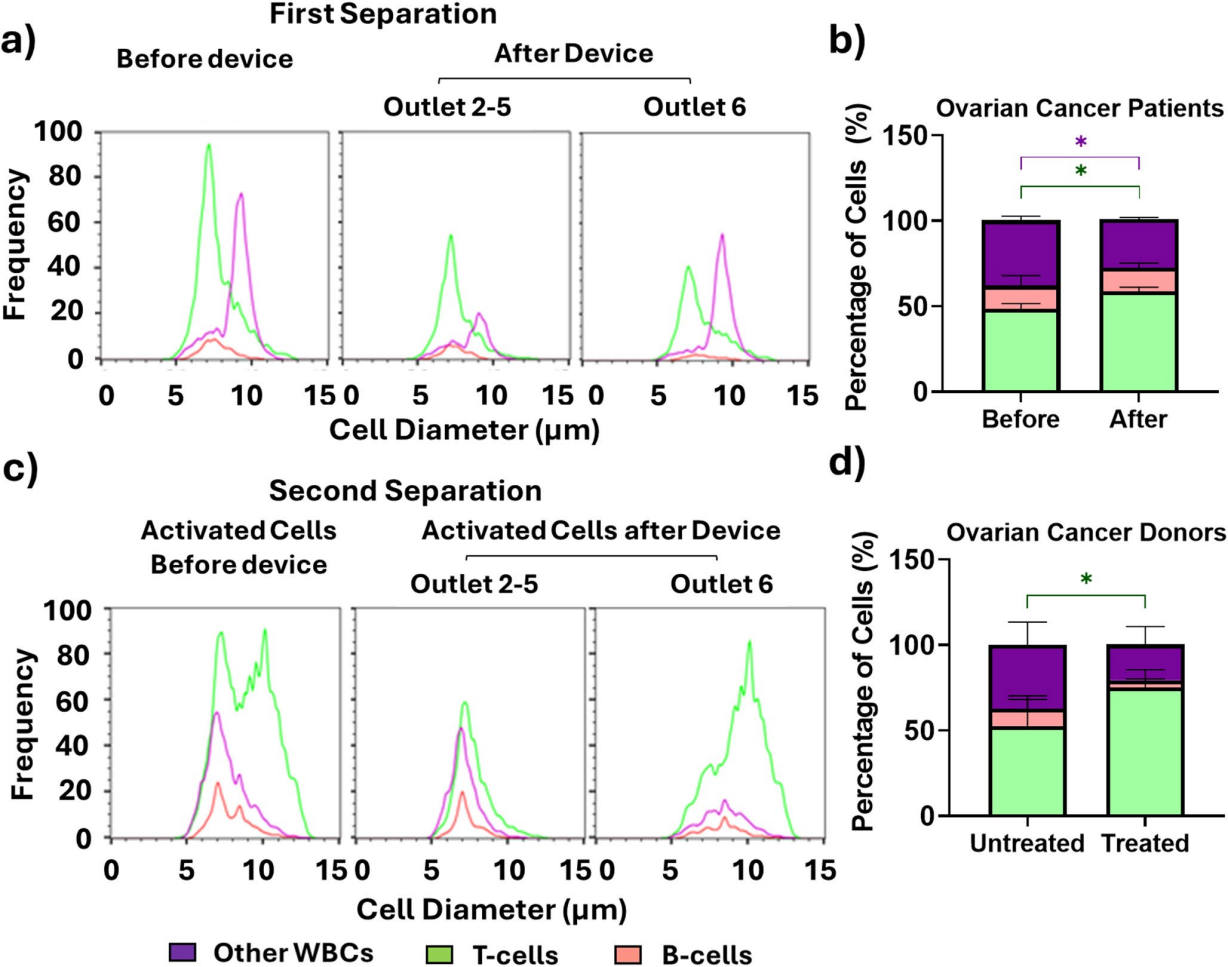
Inertial microfluidic enrichment of activated T-cells in ovarian cancer patient donors. Size distributions histograms and cell percentages of leukocyte subsets (**a** and **b**) before and after first inertial microfluidic separation and (c and d) before and after second inertial microfluidic separation following activation of T-cells. T-cells (green), B-cells (red) and other WBCs (violet). (*n* = 3 ovarian cancer patient samples, * *p* < 0.05, ** *p* < 0.005, non-parametric two- way mixed model ANOVA for matched values was used followed by Bonferroni’s multiple comparisons test)

Towards demonstrating the feasibility of our two-step enrichment methodology to real patient samples, we next tested its performance in enriching the T-cell fraction in solid cancer patient donors, namely ovarian cancer (*n* = 3 patients). Using the optimized device and operating condition, the first inertial microfluidic enrichment step yielded a lymphocyte purity of 72% (SD ± 3%) from a starting lymphocyte purity of 62% (SD ± 5%) and a T-cell purity of 59% (SD ± 4%) from a starting T-cell purity of 48% (SD ± 6%) at recoveries of 53% (SD ± 25%) and 55% (SD ± 25%) respectively. We were able to deplete 59% (SD ± 25%) of the other white blood cells as shown in Fig. 5a, b. It should be noted that the enrichment of the lymphocyte fraction in the ovarian cancer patients was not as good in the healthy donors. This reduced enrichment of the lymphocyte fraction in cancer patients has also been reported in other physical enrichment platforms as elutriation devices [13].

Next, we validated the suitability of the T-cell activation protocol selected from the healthy donor samples. We confirmed that similar size changes occurred upon activation in ovarian cancer donor PBMC samples. After two-day activation, T-cells increased in size from a mean size of 7.8 (SD ± 0.4) to 8.1 μm (SD ± 0.5), while the average size of B-cell was 7.4 μm (SD ± 0.8) and other white blood cells were 7.1 μm (SD ± 0.8). A clear change in the T cells size distribution was obtained at day two post activation as shown in Fig. 5c (activated before device) with a bimodal cell size distribution associated to the appearance of an enlarged T cell population of significantly larger size compared to B-cells and other white blood cells. We also confirmed the effect of the two-day activation on the relative percentage of different cell types. In the lymphocytic fraction enriched by the first inertial microfluidic step, the percentage of T-cells following the two-day activation step was 64% (vs. 61% at day 0), B- cells was 5% (vs. 11% at day 0) and other white blood cells was 29% (vs. 28% at day 0). This confirms that contaminating leukocytes present in the enriched lymphocyte fraction are likely to remain with T-cells in the transduction step, which may affect T-cells’ transduction, expansion and phenotype.

We next tested the enrichment of T-cells afforded by inertial microfluidic processing of the activated lymphocyte fraction. T-cells were enriched in outlet 6 reaching a purity of 70% (SD ± 10%) and a 64% recovery (SD ± 5%) as shown in Fig. 5c and d. While B-cell depletion was 58% in two of the samples, it was significantly lower in the third sample with only 20% depletion, indicating patient to patient variation. This can be linked to changes in the characteristics of the B cells depending on the type of ovarian cancer and state of the disease. For example, it has been reported that B-cells may activate and differentiate into plasma cells in some high-grade serous ovarian cancer [52].

Overall, the T-cell recovery in the two-step inertial microfluidic process was 53% (SD ± 3%) and the final T-cell purity was increased to 70%SD ± 10% vs. 52% SD ± 3% compared to the untreated two day activated as shown in Fig. 5d. The observed lower recovery of T-cells compared to the healthy donor samples can be explained by the fact that T-cells were found to be larger pre-activation in the ovarian cancer patient samples (7.8 μm SD ± 0.4 vs. 7.5 μm SD ± 0.2 for healthy donor T-cells (statistically significant, *p* < 0.05) which compromised T-cell recovery in the first separation step. This might be explained by the immunological response to the disease in these ovarian cancer patients [53, 54]. More generally, substantial variability in T-cell purification success in cancer patients is well-documented[17, 55–58].

Compared to magnetic bead selection, the dual separation microfluidic method provides the ability to enrich T-cells without the need for a de-beading step which is required with bead-based enrichment to avoid contamination of the final CAR-T product as set by the FDA guidelines [59]. It also avoids bead-associated activation of the T-cells that influences the functionality of the T-cells downstream [60]. In addition, while immunomagnetic bead-based positive selection typically achieves higher purities, it often results in reduced T-cell yields, especially in cancer patients [57]. Conversely, the microfluidic method achieves higher purity compared to negative bead selection [60]. However, to fully validate the clinical relevance of the proposed microfluidic method and assess its potential benefits, a comparative study against gold-standard negative and positive selection magnetic beads is warranted as well as testing with other types of cancers.

### Effect of two-step inertial microfluidic T-cell enrichment process on their viability, ex vivo proliferation and phenotyping

**Fig. 6:**
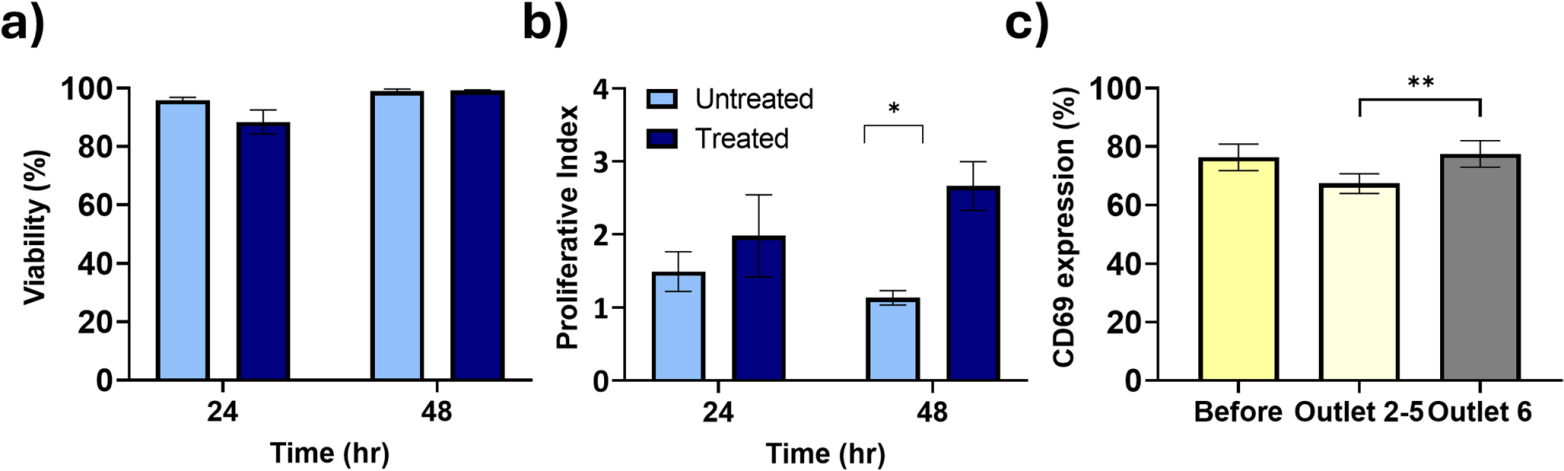
Effect of the two-step inertial microfluidic enrichment on T-cells their viability and proliferation. (**a**) Cell viability with and without two step inertial microfluidic processing as measured by PI exclusion. (**b**) Cell proliferation as tested by CFSE at 24 and 48 h post second separation (*n* = 3 * *p* < 0.05. c) Percentage of CD69 expression before and after separation in the device. (*n* = 3, * *p* < 0.05, ** *p* < 0.005, and ** *p* < 0.0005 using a two-way mixed model ANOVA)

Microfluidic processing has been previously demonstrated to be gentle process on cells [44] which was confirmed in our initial studies where we observed no detrimental effects on T-cells viability and proliferation [22, 43]. However, the two-step protocol proposed here requires further manipulation of the cells and we therefore assessed its eventual effect on T-cells functionality. Cells subjected or not to the two-step inertial microfluidic enrichment process were grown post separation and the viability and proliferation of T-cells were measured at 24 h and 48 h by PI exclusion and CFSE proliferation, as shown in Fig. 6. There was no statistically significant difference in T-cell viability at 24 and 48 h post second separation in the device (*p* < 0.05, Fig. 6a). The proliferation test showed no significant differences at 24 h, however, the enriched T-cells showed a statistically significant higher proliferation at the 48 h time-point (*p* < 0.05) compared to unprocessed ones (Fig. 6b). This observation could be due to the depletion of unactivated slow proliferating T-cells. This was further investigated by looking at the percentage of activation in the enriched fraction versus the unprocessed activated cells. While no significant difference in the percentages of activation before and after enrichment was measured (Fig. 6c), the percentage of activation was significantly higher in the enriched T-cell outlet (outlet 6) compared to the depleted outlets 2–5 (78% vs. 67%, *p* < 0.05). On the other hands, it has previously been shown that smaller sized activated T-cells have lower proliferation than larger ones [38], provides a plausible explanation for the difference in proliferation as the two-step inertial microfluidic enriches larger activated T-cells over smaller unactivated and activated ones.

**Fig. 7:**
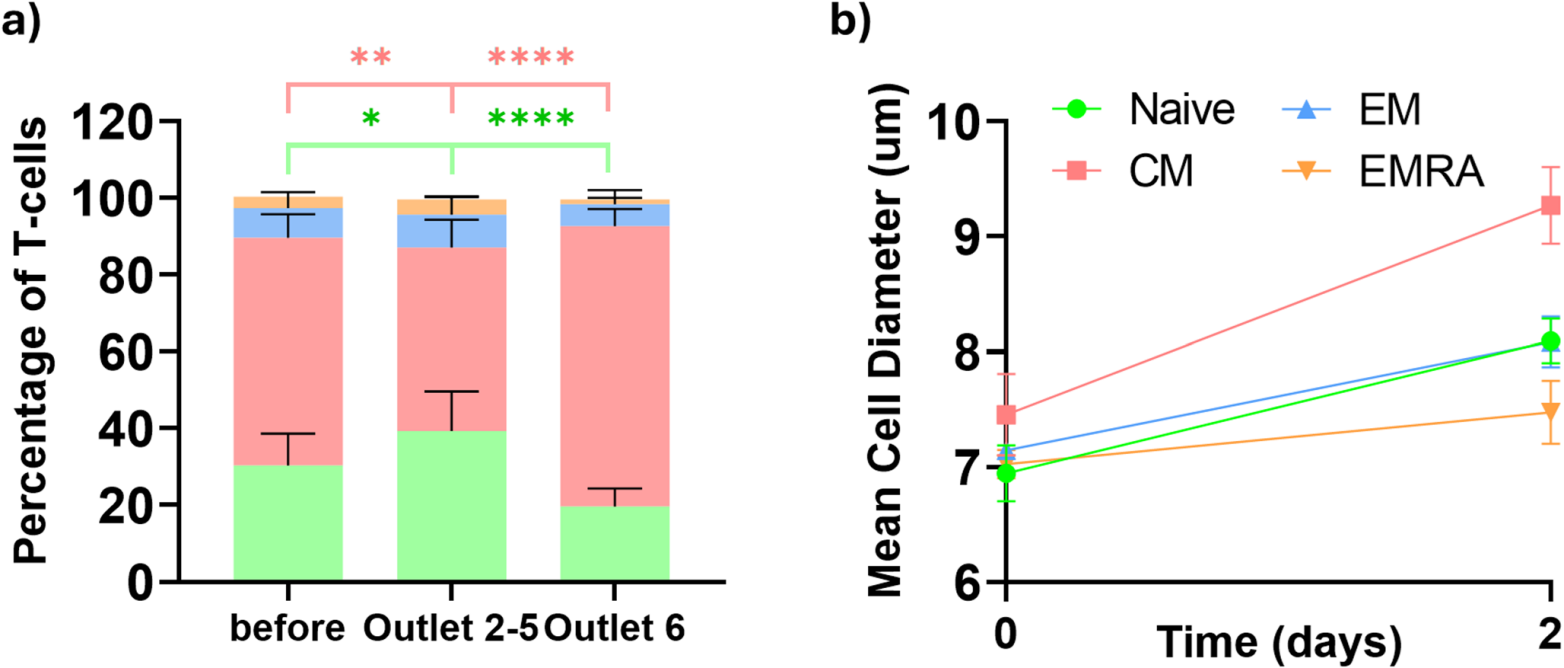
T-cell phenotyping. (**a**) Percentages of naive, central memory (CM), effector memory (EM), and terminally differentiated effector cells (EMRA) before and after microfluidic enrichment. (**b**) Cell size differences between Naïve, CM, EM and EMRAs before and 2 days after activation (*n* = 3, * *p* < 0.05, ** *p* < 0.005, and **** *p* < 0.00005 using a two-way mixed model ANOVA)

Finally, we assessed the phenotypes of the T-cells fraction upon activation and two-step enrichment, The T-cells phenotype is very important as it impacts the proliferation of the T-cells ex vivo as well as affect the in vivo T-cells performance. T-cells were differentiated into Naïve (CD45RO-, CCR7+) central memory (CD45RO+, CCR7+), effector memory (CD45RO+, CCR7-), and terminally differentiated effector EMRA T-cells (CD45RO-, CCR7-). Following the standard 2-day activation protocol, T-cells with a central memory phenotype were found to exhibit larger mean size compared to effector memory, naïve or the EMRAs T-cells as shown in Fig. 7b. This led to an enrichment of T-cells with a central memory phenotype as shown in Fig. 7a. This observation could also explain the higher proliferation of the enriched fraction as memory T-cells tend to have higher proliferation compared to naïve T-cells [61].

## Discussion

Taking advantage of the inherent enlargement of T-cells upon activation, we devised a novel two-stage T-cell enrichment approach based on inertial microfluidics. The small cellular fraction – including T- and B-lymphocytes – is enriched in the first stage of the process while the enlarged activated T-cells are enriched in the second stage at day 2. Overall, the two-stage inertial microfluidic protocol yielded T-cells purities of approximately 90% and 70% from starting purities of 45% and 48% for healthy donor and ovarian cancer patient samples respectively with overall recovery yields of 80% and 64% respectively. The two-step microfluidic enrichment had no detrimental effects on the proliferation of T-cells. In addition, we previously showed that the inertial microfluidic processing of a preclinical CAR-T cell product did not compromise the cytotoxic function or the proportion of CAR-positive T-cells [43]. While thorough assessment is warranted before clinical trial of such a methodology, we anticipate that similarly there would be no effect on the functionality CAR T cells produced using the two-step microfluidic method. Finally, the activation regime chosen here is widely utilized for specific T-cell activation in the manufacture of CAR-T cells, ensuring the compatibility of the proposed enrichment approach with contemporary protocols.

In order to assess the merit of our two-step approach, benchmarking its performance against methodologies currently used in T cell separation is important. Current industry standards include immunomagnetic bead-based selection and counterflow elutriation and in both cases, documented performance profiles have been reported.

Positive immunomagnetic selection is typically characterised by very high target cell purity, often exceeding 95 to 97% but typically at the cost of lower recoveries [66]. For example, immunomagnetic enrichment targeting CD4/CD8 T cell subsets has been reported to yield 93–95% purity with 57–68% recovery from fresh apheresis derived samples [67]. Similarly, a purities of ~ 97% with recoveries ranging from 52 to 55% have been reported with standard CD3/CD28 bead protocols from PBMC starting material [68]. The excellent purity afforded by positive immunomagnetic enrichment is a significant advantage for downstream culture uniformity but the limited recovery yield often require the use larger starting volumes or multiple rounds of processing to achieve clinically relevant cell numbers [69]. In addition other limitations of this approach include the need for bead removal to avoid residual beads in the final cell product (as per regulatory guidelines) [59, 70], potential effect on cell phenotype/activation status through bead-induced signalling [71] and the additional dead volume and processing steps introduced in closed manufacturing workflows. On the other hand, negative immunomagnetic selection typically yields lower purities ~ 78% but higher recoveries ~ 70%, illustrating the classic trade-off between purity and yield seen in conventional immunomagnetic approaches [66].

Meanwhile; counterflow elutriation is the current gold standard physical enrichment method. Elutriation has been shown to yield increased CAR-T cell yields relative to unsorted starting material. However, purities achieved are much lower than immunomagnetic based selection, ranging from 58% to 62% [72, 73]. Despite this limitation, elutriation is used in the manufacture of commercialized CAR-T cell products as Yescarta^®^ [74] and Abecma [57].

Compared to these gold-standard methods, the two-stage inertial microfluidic approach reported here offers a label free enrichment strategy that balances purity and recovery without introducing antibodies or beads. The lower purity compared to positive immunomagnetic selection may be offset by the better recovery and preservation of cell functionality as well as obviating the need for bead removal step and possible activation artefacts common with positive immunomagnetic protocols.

It is important to note that the performance varied according to the type of sample being processed, with blood samples from ovarian cancer patients yielding lower purities and yields compared to samples from healthy donors. Similar variability in the enriched T-cell purity in patient samples has been previously reported for standard methodologies [69, 70]. and is likely due to the more heterogeneous nature of real blood samples. Therefore, despite our previous demonstration that this approach can successfully deplete leukemic blasts in the first separation step to generate higher-purity T-cell populations [71] and its ability to deplete smaller sized blood cells in the second separation step as demonstrated here, further validation across a broader range of cancer types and patient-derived samples is required to fully assess the robustness of the methodology and. to determine its clinical applicability.

The performance of the microfluidic device could be further improved by various methods, for example by integrating contraction-expansion arrays [72] or by using hybrid devices integrating multiple principles in addition to inertial separation [73]. However, the inevitable overlap in physical features between T cells and contaminating cells imposes an inherent limitation to the performance of the process, especially in real cancer patient samples.

Owing to its overall good performance, robustness and integrability with current manufacturing practice, the proposed two-stage inertial microfluidic process is a promising method for T-cell enrichment. In healthy blood samples, the two-stage process yielded significantly higher T-cell enrichment yield compared to standard elutriation. In addition, microfluidic inertial cell separation offers several advantages compared to commercially available automated cell separation devices such as elutriation. It is highly flexible in term of processing sample volumes and cell numbers, and has lower dead volume which is an essential characteristic in the case of pediatric patient samples [74–76]. On the other hand, the processed sample volumes can be readily increased using a stacked configuration [77]. Another feature is the relatively lower flow rates compared to centrifugation based methods which is more gentle with cells [78, 79]. While not investigated in the present study, the two-stage inertial microfluidics process also offer opportunities to significantly deplete residual red blood cells [80].

A critical consideration for the implementation of a T-cell enrichment technology in the CAR T cells manufacture is its ability to handle the volume required for preparing clinical doses. Inertial microfluidic chips such as the one used in this proof-of-principle study have relatively high throughputs being able to process up to 5 × 106 cells/min [22]. The chip used can process 1 mL/min. However, a typical leukapheresis bag contains up to 9 × 10^9^ white blood cells in up to a 200 mL volume [81]. In order to be of the correct concentration for the operation of the chip, the volume will have to be increased to about 2 L. If only one chip is used it would take about 33 h which is not feasible in a typical clinical setting. However, improved designs and advances in the manufacture of these microfluidic chips could substantially increase the throughput. For example, stacking of multiple microfluidic chips is feasible and would likely enable processing clinically relevant volumes. The stacking of microfluidic devices has been successfully demonstrated, resulting in about 15 fold decrease in processing time in inertial microfluidics [44] and enabling successful use of acoustophoresis microfluidic chips in CAR-T cell manufacture [30, 82].

Implementation of inertial microfluidic cell separation could however assist in reducing the costs of CAR-T cell manufacture as well as circumvent the drawbacks associated to immunomagnetic selection. However, similar to other T-cells enrichment/selection methods, the performance is inevitably affected by the type of cancer, stage of the disease and immunological variability among patients. Further validation in different types of cancer is necessary to fully evaluate the significance of the inertial microfluidic enrichment of activated T-cells.

## Conclusion

In this research a two stage inertial microfluidic enrichment process utilised activation induced T cell enlargement to achieve a label free T cell isolation with purities of 90% in healthy donor samples and 70% in ovarian cancer samples at acceptable recoveries. The proliferative and functional capacity of the cells were preserved and being compatible with standard activation protocols, this method offers practical advantages such as flexibility in sample volume, low dead volume and gentle processing conditions. Given its integrability with existing manufacturing workflows and potential to reduce dependence on expensive immunomagnetic selection, further validation with diverse cancer samples is warranted to establish the translational significance of this approach.

## Data Availability

No datasets were generated or analysed during the current study.

## References

[1] Schuster SJ, Bishop MR, Tam CS, Waller EK, Borchmann P, McGuirk JP, et al. Tisagenlecleucel in adult relapsed or refractory diffuse large b-cell lymphoma. N Engl J Med. 2019;380:45–56. 10.1056/NEJMoa1804980. 30501490 10.1056/NEJMoa1804980

[2] Locke FL, Neelapu SS, Bartlett NL, Siddiqi T, Chavez JC, Hosing CM, et al. Phase 1 results of ZUMA-1: a multicenter study of KTE-C19 anti-CD19 CAR T cell therapy in refractory aggressive lymphoma. Mol Ther. 2017;25:285–95. 10.1016/j.ymthe.2016.10.020. 28129122 10.1016/j.ymthe.2016.10.020 PMC5363293

[3] Hildreth C. History of CAR-T Cell Therapy Spans 60 + Years [Internet]. BioInformant. 2020 [cited 2020 Sept 28]. https://bioinformant.com/history-of-car-t-cell-therapy/. Accessed 28 Sept 2020.

[4] Iyer RK, Bowles PA, Kim H, Dulgar-Tulloch A. Industrializing autologous adoptive immunotherapies: manufacturing advances and challenges. Front Med. 2018;5:150. 10.3389/fmed.2018.00150. 10.3389/fmed.2018.00150 PMC5974219 29876351

[5] Wang X, Rivière I. Clinical manufacturing of CAR T cells: foundation of a promising therapy. Molecular Therapy - Oncolytics. 2016;3:16015. 10.1038/mto.2016.15. 27347557 10.1038/mto.2016.15 PMC4909095

[6] Ran T, Eichmüller SB, Schmidt P, Schlander M. Cost of decentralized CAR T-cell production in an academic nonprofit setting. Int J Cancer. 2020. 10.1002/ijc.33156. 32535920 10.1002/ijc.33156

[7] Harrison RP, Ruck S, Rafiq QA, Medcalf N. Decentralised manufacturing of cell and gene therapy products: learning from other healthcare sectors. Biotechnol Adv. 2018;36:345–57. 10.1016/j.biotechadv.2017.12.013. 29278756 10.1016/j.biotechadv.2017.12.013

[8] Shah M, Krull A, Odonnell L, de Lima MJ, Bezerra E. Promises and challenges of a decentralized CAR T-cell manufacturing model. Front Transplant. 2023. 10.3389/frtra.2023.1238535. 38993860 10.3389/frtra.2023.1238535 PMC11235344

[9] Egri N, Ortiz de Landazuri I, San Bartolomé C, Ortega JR, Español-Rego M, Juan M. Cart manufacturing process and reasons for academy-pharma collaboration. Immunol Lett. 2020;217:39–48. 10.1016/j.imlet.2019.10.014. 31669547 10.1016/j.imlet.2019.10.014

[10] Huang R, Li X, He Y, Zhu W, Gao L, Liu Y, et al. Recent advances in CAR-T cell engineering. Journal of Hematology & Oncology. 2020;13:86. 10.1186/s13045-020-00910-5. 32616000 10.1186/s13045-020-00910-5 PMC7333410

[11] Nam S, Smith J, Yang G. Driving the next wave of innovation in CAR T-cell therapies | McKinsey [Internet]. 2019 [cited 2020 Sept 14]. https://www.mckinsey.com/industries/pharmaceuticals-and-medical-products/our-insights/driving-the-next-wave-of-innovation-in-car-t-cell-therapies. Accessed 14 Sept 2020.

[12] Wall DA, Krueger J. Chimeric antigen receptor T cell therapy comes to clinical practice. Curr Oncol. 2020;27:S115–23. 10.3747/co.27.5283. 32368181 10.3747/co.27.5283 PMC7193999

[13] Stroncek DF, Lee DW, Ren J, Sabatino M, Highfill S, Khuu H, et al. Elutriated lymphocytes for manufacturing chimeric antigen receptor T cells. J Transl Med. 2017;15:59. 10.1186/s12967-017-1160-5. 28298232 10.1186/s12967-017-1160-5 PMC5353875

[14] Ribickas AJ, Thigpen SL, Janssen W, Kelley L. An automated method of enrichment of lymphocytes from apheresis products for cellular engineering. Cytotherapy Elsevier. 2018;20:S70. 10.1016/j.jcyt.2018.02.197.

[15] Mock U, Nickolay L, Philip B, Cheung GW-K, Zhan H, Johnston ICD, et al. Automated manufacturing of chimeric antigen receptor T cells for adoptive immunotherapy using clinimacs prodigy. Cytotherapy. 2016;18:1002–11. 10.1016/j.jcyt.2016.05.009. 27378344 10.1016/j.jcyt.2016.05.009

[16] Tostoes R, Zhang C, Saloio J, Cushman J, Cushing K, Barber A, et al. Acoustic affinity cell selection: a non-paramagnetic scalable technology for T cell selection from unprocessed apheresis products. Cytotherapy Elsevier. 2020;22:S16. 10.1016/j.jcyt.2020.03.482.

[17] Turtle CJ, Hanafi L-A, Berger C, Gooley TA, Cherian S, Hudecek M, et al. CD19 CAR–T cells of defined CD4+:CD8 + composition in adult B cell ALL patients. J Clin Invest. 2016;126:2123–38. 10.1172/JCI85309. 27111235 10.1172/JCI85309 PMC4887159

[18] Wang X, Stefanski J, Borquez-Ojeda O, Qu J, Hack A, He Q, et al. Comparison of CTS^™^ Dynabeads^®^ CD3/CD28, Miltenyi transact CD3/28 and expact beads for large-scale CAR T cell manufacturing. Hum Gene Ther. 2015;26:A31.

[19] Pierzchalski A, Mittag A, Bocsi J, Tarnok A, Public Library of Science. An innovative cascade system for simultaneous separation of multiple cell types. PLoS One. 2013;8:e74745. 10.1371/journal.pone.0074745. 24040334 10.1371/journal.pone.0074745 PMC3765397

[20] Li Y, Kurlander RJ. Comparison of anti-CD3 and anti-CD28-coated beads with soluble anti-CD3 for expanding human T cells: differing impact on CD8 T cell phenotype and responsiveness to restimulation. J Transl Med. 2010;8:104. 10.1186/1479-5876-8-104. 20977748 10.1186/1479-5876-8-104 PMC2987859

[21] Dias J, Garcia J, Agliardi G, Roddie C. CAR-T cell manufacturing landscape-lessons from the past decade and considerations for early clinical development. Molecular Therapy - Methods & Clinical Development. 2024;32:101250. 10.1016/j.omtm.2024.101250. 38737799 10.1016/j.omtm.2024.101250 PMC11088187

[22] Elsemary MT, Maritz MF, Smith LE, Warkiani ME, Thierry B. Enrichment of T-lymphocytes from leukemic blood using inertial microfluidics toward improved chimeric antigen receptor-T cell manufacturing. Cytotherapy. 2024. 10.1016/j.jcyt.2024.05.005. 38819362 10.1016/j.jcyt.2024.05.005

[23] Warkiani ME, Guan G, Luan KB, Lee WC, Bhagat AAS, Chaudhuri PK, et al. Slanted spiral microfluidics for the ultra-fast, label-free isolation of Circulating tumor cells. Lab Chip. 2013;14:128–37. 10.1039/C3LC50617G. 10.1039/c3lc50617g 23949794

[24] Zhang J, Yuan D, Sluyter R, Yan S, Zhao Q, Xia H, et al. High-throughput separation of white blood cells from whole blood using inertial microfluidics. IEEE Trans Biomed Circuits Syst. 2017;11(6):1422–1430. 10.1109/TBCAS.2017.2735440. 28866599 10.1109/TBCAS.2017.2735440

[25] Lee C-H, Bose S, Van Vliet KJ, Karp JM, Karnik R. Examining the lateral displacement of HL60 cells rolling on asymmetric P-selectin patterns. Langmuir. 2011;27:240–9. 10.1021/la102871m. 21141947 10.1021/la102871m PMC3068857

[26] Gou Y, Jia Y, Wang P, Sun C. Progress of inertial microfluidics in principle and application. Sensors (Basel). 2018;18(6):1762. 10.3390/s18061762. 29857563 10.3390/s18061762 PMC6021949

[27] Zhang J, Li W, Alici G. Inertial microfluidics: mechanisms and applications. In: Zhang D, Wei B, editors. Advanced mechatronics and MEMS devices II [Internet]. Cham: Springer International Publishing; 2017. pp. 563–93. [cited 2017 Sept 18]. 10.1007/978-3-319-32180-6_25.

[28] Campos-González R, Skelley AM, Gandhi K, Inglis DW, Sturm JC, Civin CI, et al. Deterministic lateral displacement: the next-generation CAR T-cell processing? SLAS Technology. 2018;23:338–51. 10.1177/2472630317751214. 29361868 10.1177/2472630317751214

[29] Read 2 Min. GPB Scientific Awarded Phase II of $2.2 M NIH Grant to Complete Development of Microfluidic CAR-T Cell Processing Device [Internet]. BioSpace. 2019 [cited 2025 Dec 14]. https://www.biospace.com/gpb-scientific-awarded-phase-ii-of-2-2m-nih-grant-to-complete-development-of-microfluidic-car-t-cell-processing-device. Accessed 14 Dec 2025.

[30] Draper. Using Precision Engineering to Streamline Manufacturing of Next-Generation Cellular Therapies [Internet]. Draper. 2019 [cited 2020 Apr 12]. https://www.draper.com/news-releases/using-precision-engineering-streamline-manufacturing-next-generation-cellular. Accessed 12 Apr 2020.

[31] Moore N, Chevillet JR, Healey LJ, McBrine C, Doty D, Santos J, et al. A microfluidic device to enhance viral transduction efficiency during manufacture of engineered cellular therapies. Sci Rep Nat Publishing Group. 2019;9:1–11. 10.1038/s41598-019-50981-9. 10.1038/s41598-019-50981-9 PMC6806008 31641163

[32] Aranda Hernandez J, Heuer C, Bahnemann J, Szita N. Microfluidic devices as process development tools for cellular therapy manufacturing. In: Bahnemann J, Grünberger A, editors. Microfluidics in biotechnology [Internet]. Cham: Springer International Publishing; 2022. pp. 101–27. [cited 2024 Apr 3]. 10.1007/10_2021_169.

[33] Halldorsson S, Lucumi E, Gómez-Sjöberg R, Fleming RMT. Advantages and challenges of microfluidic cell culture in polydimethylsiloxane devices. Biosens Bioelectron. 2015;63:218–31. 10.1016/j.bios.2014.07.029. 25105943 10.1016/j.bios.2014.07.029

[34] PhD JDG. In Your Head: Ori biotech is designing modules for scalable cell therapy manufacturing [Internet]. GEN - Genetic Engineering and Biotechnology News. 2023 [cited 2024 Sept 9]. https://www.genengnews.com/topics/translational-medicine/in-your-head-ori-biotech-is-designing-modules-for-scalable-cell-therapy-manufacturing/. Accessed 9 Sept 2024.

[35] Cellares. Cell_Shuttle_End-to-End_Manufacturing_v1.1.pdf [Internet]. [cited 2024 Sept 9]. https://www.cellares.com/wp-content/uploads/Cell_Shuttle_End-to-End_Manufacturing_v1.1.pdf. Accessed 9 Sept 2024.

[36] Chiaretti S, Zini G, Bassan R. Diagnosis and subclassification of acute lymphoblastic leukemia. Mediterr J Hematol Infect Dis. 2014. 10.4084/MJHID.2014.073. 25408859 10.4084/MJHID.2014.073 PMC4235437

[37] King JF, Lam JT. A practical approach to diagnosis of B-cell lymphomas with diffuse large cell morphology. Arch Pathol Lab Med. 2020;144:160–7. 10.5858/arpa.2019-0182-RA. 31990228 10.5858/arpa.2019-0182-RA

[38] Pollizzi KN, Waickman AT, Patel CH, Sun IH, Powell JD. Cellular size as a means of tracking mTOR activity and cell fate of CD4 + T cells upon antigen recognition. PLoS One. 2015. 10.1371/journal.pone.0121710. 25849206 10.1371/journal.pone.0121710 PMC4388710

[39] Iritani BM, Delrow J, Grandori C, Gomez I, Klacking M, Carlos LS, et al. Modulation of T-lymphocyte development, growth and cell size by the Myc antagonist and transcriptional repressor Mad1. EMBO J. 2002;21:4820–30. 10.1093/emboj/cdf492. 12234922 10.1093/emboj/cdf492 PMC126288

[40] Huang Y, Wang X-B, Gascoyne PRC, Becker FF. Membrane dielectric responses of human T-lymphocytes following mitogenic stimulation. Biochimica et Biophysica Acta (BBA) - Biomembranes. 1999;1417:51–62. 10.1016/S0005-2736(98)00253-3. 10076035 10.1016/s0005-2736(98)00253-3

[41] Elsemary MT, Maritz MF, Smith LE, Warkiani M, Bandara V, Napoli S, et al. Inertial microfluidic purification of CAR-T-cell products. Adv Biol. 2022;6(1):2101018. 10.1002/adbi.202101018. 10.1002/adbi.202101018 34881810

[42] Warkiani ME, Khoo BL, Tan D-W, Bhagat AAS, Lim W-T, Yap YS, et al. An ultra-high-throughput spiral microfluidic biochip for the enrichment of circulating tumor cells. Analyst. 2014;139:3245–55. 10.1039/c4an00355a. 24840240 10.1039/c4an00355a

[43] Simms PE, Ellis TM. Utility of flow cytometric detection of CD69 expression as a rapid method for determining poly- and oligoclonal lymphocyte activation. Clin Diagn Lab Immunol. 1996;3:301–4. 8705673 10.1128/cdli.3.3.301-304.1996 PMC170336

[44] Ghamlouch H, Ouled-Haddou H, Guyart A, Regnier A, Trudel S, Claisse J-F, et al. Phorbol myristate acetate, but not CD40L, induces the differentiation of CLL B cells into Ab-secreting cells. Immunol Cell Biol. 2014;92:591–604. 10.1038/icb.2014.37. 24797583 10.1038/icb.2014.37 PMC4134517

[45] Yanaba K, Bouaziz J-D, Matsushita T, Tsubata T, Tedder TF. The development and function of regulatory B cells expressing IL-10 (B10 cells) requires antigen receptor diversity and TLR signals. J Immunol. 2009;182:7459–72. 10.4049/jimmunol.0900270. 19494269 10.4049/jimmunol.0900270 PMC3733128

[46] Xu H, Liew LN, Kuo IC, Huang CH, Goh DL-M, Chua KY. The modulatory effects of lipopolysaccharide-stimulated B cells on differential T-cell polarization. Immunology. 2008;125:218–28. 10.1111/j.1365-2567.2008.02832.x. 18355243 10.1111/j.1365-2567.2008.02832.x PMC2561127

[47] Dan L, Kang-Zheng L. Optimizing viral transduction in immune cell therapy manufacturing: key process design considerations. J Transl Med. 2025;23:501. 10.1186/s12967-025-06524-0. 40316943 10.1186/s12967-025-06524-0 PMC12046913

[48] Cribbs AP, Kennedy A, Gregory B, Brennan FM. Simplified production and concentration of lentiviral vectors to achieve high transduction in primary human T cells. BMC Biotechnol. 2013;13:98. 10.1186/1472-6750-13-98. 24215295 10.1186/1472-6750-13-98 PMC3830501

[49] Powell DJ, Brennan AL, Zheng Z, Huynh H, Cotte J, Levine BL. Efficient clinical-scale enrichment of lymphocytes for use in adoptive immunotherapy using a modified counterflow centrifugal elutriation program. Cytotherapy Elsevier. 2009;11:923–35. 10.3109/14653240903188921. 10.3109/14653240903188921 19903104

[50] Montfort A, Pearce O, Maniati E, Vincent BG, Bixby L, Böhm S, et al. A strong B-cell response is part of the immune landscape in human high-grade serous ovarian metastases. Clin Cancer Res. 2017;23:250–62. 10.1158/1078-0432.CCR-16-0081. 27354470 10.1158/1078-0432.CCR-16-0081 PMC5928522

[51] Salas-Benito D, Vercher E, Conde E, Glez-Vaz J, Tamayo I, Hervas-Stubbs S. Inflammation and immunity in ovarian cancer. Eur J Cancer Suppl. 2020;15:56–66. 10.1016/j.ejcsup.2019.12.002. 10.1016/j.ejcsup.2019.12.002 PMC7569134 33240443

[52] Nelson BH. The impact of T-cell immunity on ovarian cancer outcomes. Immunol Rev. 2008;222:101–16. 10.1111/j.1600-065X.2008.00614.x. 18363996 10.1111/j.1600-065X.2008.00614.x

[53] Ad F. The challenge of variability in chimeric antigen receptor t cell manufacturing. Regen Eng Transl Med. 2020. 10.1007/s40883-019-00124-3. [cited 2023 Sept 5];6. Regenerative engineering and translational medicine [Internet]. 10.1007/s40883-019-00124-3 PMC7728294 33313382

[54] Ruella M, Xu J, Barrett DM, Fraietta JA, Reich TJ, Ambrose DE, et al. Induction of resistance to chimeric antigen receptor T cell therapy by transduction of a single leukemic B cell. Nat Med. 2018;24:1499–503. 10.1038/s41591-018-0201-9. 30275568 10.1038/s41591-018-0201-9 PMC6511988

[55] Lock D, Mockel-Tenbrinck N, Drechsel K, Barth C, Mauer D, Schaser T, et al. Automated manufacturing of potent CD20-directed chimeric antigen receptor T cells for clinical use. Hum Gene Ther. 2017;28:914–25. 10.1089/hum.2017.111. 28847167 10.1089/hum.2017.111

[56] Pierzchalski A, Mittag A, Bocsi J, Tarnok A. An innovative cascade system for simultaneous separation of multiple cell types. PLoS One. 2013;8:e74745. 10.1371/journal.pone.0074745. 24040334 10.1371/journal.pone.0074745 PMC3765397

[57] Ayala Ceja M, Khericha M, Harris CM, Puig-Saus C, Chen YY. CAR-T cell manufacturing: major process parameters and next-generation strategies. J Exp Med. 2024;221:e20230903. 10.1084/jem.20230903. 38226974 10.1084/jem.20230903 PMC10791545

[58] Song HW, Benzaoui M, Dwivedi A, Underwood S, Shao L, Achar S, et al. Manufacture of CD22 CAR t cells following positive versus negative selection results in distinct cytokine secretion profiles and γδ t cell output. Molecular Therapy - Methods & Clinical Development. 2024. 10.1016/j.omtm.2023.101171. 10.1016/j.omtm.2023.101171 PMC10827561 38298420

[59] Berard M, Tough DF. Qualitative differences between naïve and memory T cells. Immunology. 2002;106(2):127–38. 10.1046/j.1365-2567.2002.01447.x. 12047742 10.1046/j.1365-2567.2002.01447.x PMC1782715

[60] Song HW, Benzaoui M, Dwivedi A, Underwood S, Shao L, Achar S, et al. Manufacture of CD22 CAR T cells following positive versus negative selection results in distinct cytokine secretion profiles and γδ T cell output. Mol Ther Methods Clin Dev. 2023;32:101171. 10.1016/j.omtm.2023.101171. 38298420 10.1016/j.omtm.2023.101171 PMC10827561

[61] Langa P, Sharma K, Sellers DL, Placencia V, Smith EA, Fick D, et al. Enrichment of CD4 + and CD8 + T lymphocytes with a column-free flow-based device for clinical cell manufacturing. Cytotherapy. 2025;27:534–43. 10.1016/j.jcyt.2024.12.009. 39891633 10.1016/j.jcyt.2024.12.009

[62] CD3/CD28 + T cell Isolation [Internet]. Applied Cells. [cited 2025 Dec 14]. https://appliedcells.com/cell-therapy/cd3-cd28-t-cell-isolation/. Accessed 14 Dec 2025.

[63] Song HW, Benzaoui M, Dwivedi A, Underwood S, Shao L, Achar S, et al. <article-title update="added">Manufacture of CD22 CAR T cells following positive versus negative selection results in distinct cytokine secretion profiles and γδ T cell output. Mol Ther Methods Clin Dev. 2024;32:101171. 10.1016/j.omtm.2023.101171. 38298420 10.1016/j.omtm.2023.101171 PMC10827561

[64] van der Walle CF, Godbert S, Saito G, Azhari Z. Formulation considerations for autologous T cell drug products. Pharmaceutics. 2021;13:1317. 10.3390/pharmaceutics13081317. 34452278 10.3390/pharmaceutics13081317 PMC8400304

[65] González KMP, Tran LNT, Iyer PR, Wu X, Choe H, Rezaei B, et al. Blood cell separation with magnetic techniques: a critical review. Lab Chip. 2025;25:2521–65. 10.1039/D5LC00180C. 40356397 10.1039/d5lc00180c

[66] Weiss R, Gerdes W, Berthold R, Sack U, Koehl U, Hauschildt S, et al. Comparison of three CD3-specific separation methods leading to labeled and label-free T cells. Cells. 2021;10:2824. 10.3390/cells10112824. 34831046 10.3390/cells10112824 PMC8616525

[67] Stroncek DF, Lee DW, Ren J, Sabatino M, Highfill S, Khuu H, et al. Elutriated lymphocytes for manufacturing chimeric antigen receptor T cells. J Transl Med. 2017;15:59. 10.1186/s12967-017-1160-5. 28298232 10.1186/s12967-017-1160-5 PMC5353875

[68] yescarta-epar-public. -assessment-report_en.pdf [Internet]. [cited 2025 Dec 14]. https://www.ema.europa.eu/en/documents/assessment-report/yescarta-epar-public-assessment-report_en.pdf. Accessed 14 Dec 2025.

[69] Baguet C, Larghero J, Mebarki M. Early predictive factors of failure in autologous CAR T-cell manufacturing and/or efficacy in hematologic malignancies. Blood Adv. 2024;8:337–42. 10.1182/bloodadvances.2023011992. 38052048 10.1182/bloodadvances.2023011992 PMC10788849

[70] Lock D, Mockel-Tenbrinck N, Drechsel K, Barth C, Mauer D, Schaser T, et al. Automated manufacturing of potent CD20-directed chimeric antigen receptor T cells for clinical use. Hum Gene Ther. 2017;28:914–25. 10.1089/hum.2017.111. 28847167 10.1089/hum.2017.111

[71] Elsemary MT, Maritz MF, Smith LE, Warkiani ME, Thierry B. Enrichment of T-lymphocytes from leukemic blood using inertial microfluidics toward improved chimeric antigen receptor-T cell manufacturing. Cytotherapy Elsevier. 2024;26:1264–74. 10.1016/j.jcyt.2024.05.005. 10.1016/j.jcyt.2024.05.005 38819362

[72] Shahraki ZH, Navidbakhsh M, Taylor RA. Coupling contraction-expansion arrays with spiral microchannels to enhance microfluidic-based particle/cell separation. Int J Comput Fluid Dynamics IAHR Website. 2022;36:63–90. 10.1080/10618562.2022.2053114.

[73] Lu Y, Ying J, Mu S, Tan W, Zhu G. Sheathless and high-throughput separation of multi-target particles combining inertia and deterministic lateral displacement (DLD) in a microchannel. Sep Purif Technol. 2024;345:127369. 10.1016/j.seppur.2024.127369.

[74] Iqbal M, Mukhamedshin A, Lezzar DL, Abhishek K, McLennan AL, Lam FW, et al. Recent advances in microfluidic cell separation to enable centrifugation-free, low extracorporeal volume leukapheresis in pediatric patients. Blood Transfus [Internet]. 2023;21:494–513. 10.2450/BloodTransfus.506. [cited 2024 Apr 5];. 37146298 10.2450/BloodTransfus.506 PMC10645346

[75] Li A, Kusuma GD, Driscoll D, Smith N, Wall DM, Levine BL, et al. Advances in automated cell washing and concentration. Cytotherapy. 2021;23:774–86. 10.1016/j.jcyt.2021.04.003. 34052112 10.1016/j.jcyt.2021.04.003

[76] Runco DV, Josephson CD, Raikar SS, Goldsmith KC, Lew G, Pauly M, et al. Hyperleukocytosis in infant acute leukemia: a role for manual exchange transfusion for leukoreduction. Transfusion. 2018;58:1149–56. 10.1111/trf.14512. 29399859 10.1111/trf.14512 PMC5912979

[77] Khoo BL, Warkiani ME, Tan DS-W, Bhagat AAS, Irwin D, Lau DP et al. Clinical Validation of an Ultra High-Throughput Spiral Microfluidics for the Detection and Enrichment of Viable Circulating Tumor Cells. Wanjin H, editor. PLoS ONE [Internet]. 2014 [cited 2024 Apr 5];9:e99409. 10.1371/journal.pone.0099409 10.1371/journal.pone.0099409 PMC4085042 24999991

[78] CTS Rotea Counterflow Centrifugation System Specifications. - AU [Internet]. [cited 2024 Apr 9]. https://www.thermofisher.com/au/en/home/clinical/cell-gene-therapy/cell-therapy/cell-therapy-manufacturing-solutions/rotea-counterflow-centrifugation-system/specifications.html. Accessed 9 Apr 2024.

[79] Carvell T, Burgoyne P, Milne L, Campbell JDM, Fraser AR, Bridle H, et al. Human leucocytes processed by fast-rate inertial microfluidics retain conventional functional characteristics. Journal of The Royal Society Interface. 2024;21:20230572. 10.1098/rsif.2023.0572. 38442860 10.1098/rsif.2023.0572 PMC10914517

[80] Wu L, Guan G, Hou HW, Bhagat AAS, Han J. Continuous rbc removal using spiral microchannel with trapezoid cross-Sect. 2012.

[81] Carbonell D, Monsalvo S, Catalá E, Pérez-Corral A, Villegas C, Falero C, et al. Advantages of high cell concentration prior to cryopreservation of initial leukapheresis in CAR-T cell therapy. Blood Transfus [Internet]. 2024;22:239–45. 10.2450/BloodTransfus.542. [cited 2025 Mar 12];. 38063787 10.2450/BloodTransfus.542 PMC11073629

[82] Dubay R, Lissandrello C, Swierk P, Moore N, Doty D, Fiering J. Scalable high-throughput acoustophoresis in arrayed plastic microchannels. Biomicrofluidics. 2019;13:034105. 10.1063/1.5096190. 31123537 10.1063/1.5096190 PMC6509045

